# Correction to Nonradiative Energy Migration in Spherical Nanoparticles Theoretical Model and Monte Carlo Study

**DOI:** 10.1021/acs.jpcc.2c06113

**Published:** 2022-09-08

**Authors:** Leszek Kułak, Agnieszka Schlichtholz, Piotr Bojarski

1. The subscript in the condition for the rate constant following eq 1 on page 11211 should be written as follows:
  where δ_*ij*_ is a Kronecker delta and .
  This formal condition for  excludes self-transfer.
2. Page 11215, second line below eq A2:
  On the right side of the relation for *B*_2_^*SD*^(ϵ), the parentheses should appear for the sake of clarity.
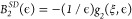
3. Page 11215, the line following eq A5:
  On the right side of the relation for *B*_3_^*SD*^(ϵ), for the sake of clarity, the parentheses should appear. Additionally a “–” sign was missing.
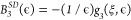
4. Page 11216, two lines before eq A11:
  The subscript for the transfer rate  should take the following proper form .

